# Editorial: Neural Mechanisms of Perceptual-Cognitive Expertise in Elite Performers

**DOI:** 10.3389/fnhum.2022.923816

**Published:** 2022-05-16

**Authors:** Daniel M. Laby, Lawrence Gregory Appelbaum, Thorben Hülsdünker, David Putrino

**Affiliations:** ^1^ChampionsEdge, LLC, New York, NY, United States; ^2^Department of Psychiatry, University of California, San Diego, San Diego, CA, United States; ^3^Department of Exercise and Sport Science, Lunex University, Differdange, Luxembourg; ^4^Luxembourg Health and Sport Science Research Institute (LHSSRI), Differdange, Luxembourg; ^5^Department of Rehabilitation and Human Performance, Icahn School of Medicine at Mount Sinai, New York, NY, United States

**Keywords:** neural, mechanism, perceptual, cognitive, elite, performers, expertise

Perception of sensory information and accompanying cognitive processing are crucial for human interactions with the environment. Elite performers in a number of human endeavors such as athletics, piloting, and surgery are exposed to extremely challenging environmental conditions resulting in the development of exceptional perceptual-cognitive skills. Although it is unknown if these abilities are primary, or are secondary to training and experience, when compared to non-experts they are superior. These enhanced abilities include greater sensitivity and reaction to visual and auditory stimuli, more rapid decision making and faster initiation of motor responses. The increased interest in, and the study of, sports and high-performance activities have enabled the development of an ever-increasing number of tools and techniques to study, quantify, evaluate, and potentially train these skills.

With this approach, elite human performance can be studied not only holistically, but also by examining the contribution of each portion of the sensory-motor pathway to decision making and action. In addition to the sensory organs, the central nervous system is integral to this process, and with increasing skill and expertise, structural and functional reorganization is noted within the central nervous system. Our ability to measure these changes provides scientists and clinicians the information necessary to identify targets and the methods to optimize training for maximal performance.

The Frontiers Research Topic entitled “*Neural Mechanisms of Perceptual-Cognitive Expertise in Elite Performers*” is aimed at further exploring the mechanisms and processes that are responsible for perceptual-cognitive expertise in elite performers. Across the seven articles there are a diverse group of reports—ranging from the specific use of auditory stimulation to increase arousal, to broader reports involving surgeons, athletes, and eGamers.

The articles in this Research Topic cover three themes. Two of the articles evaluate the role of auditory stimulation to enhance performance in Badminton and Cycling. A second set of articles look at the role of oculomotor learning and behavior in video gamers and bowling, respectively. The final set of three articles review and evaluate models by which the visual-cognitive system can be trained and enhanced through theoretical and experimental perspectives in surgeons, ice hockey, and motorsport athletes.

The article, “*Music Augmented With Isochronic Auditory Beats or Vibrotactile Stimulation Does Not Affect Subsequent Ergometer Cycling Performance: A Pilot Study*” by Fry et al. evaluates two commercially available methods and a self-selected music approach, to quantify ergogenic effects on six male and five female cyclists. The authors evaluated a variety of measurable outcomes including power output as well as felt arousal and feeling scores. The authors found no significant difference between the methods.

A second article addressing auditory stimulation entitled “*Auditory Information Accelerates the Visuomotor Reaction Speed of Elite Badminton Players in Multisensory Environments*,” by Hülsdünker et al. presents results of a comparison between monosensory and multisensory cues in 19 elite badminton athletes. The authors recorded visual and auditory sequences on a live badminton court and then isolated one from the other. These stimuli were then presented in a lab-based reaction test where athletes responded to monosensory (vision or sound) or multisensory (audio-visual) stimulation while brain activity was recorded using EEG. They find that multisensory stimuli lead to the fastest reaction times, followed by mono auditory, then mono visual stimuli. Faster reactions were further paralleled by lower latencies of visual and auditory-evoked potentials. Their results emphasize the contribution of auditory information to elite athlete performance in multisensory environments and suggest that realistic auditory stimulation may be a promising target for training in sports.

The next set of papers consider oculomotor behavior and motor learning in sports. In the publication “*Oculomotor Behavior Predict Professional Cricket Batting and Bowling Performance*” by Murray et al., the authors study 59 male T20 professional cricket athletes. The authors evaluated several measures of oculomotor movement using eye-tracking tests and compared these through multiple regression analyses to cricket performance variables. The results demonstrated predictive relationships between the eye tracking metrics and batting statistics, supporting previously published literature regarding the use of eye-tracking in sports performance evaluation.

A second article addressing oculomotor control, entitled “*Long-Term Motor Learning in the “Wild” With High Volume Video Game Data*” was authored by Listman et al. While most previously published studies in this area are limited by small sample sizes and performed over a short period of study, this study employed a very large sample (7,174 subjects) over a period of several months. The authors found improvements in performance accuracy (modest) as well as motor acuity (considerable). The greatest improvements in motor acuity were noted with an hour of practice in ecologically valid conditions, with 90% of the learning benefit after 30 min of practice per day. These results provide a proof-of-concept for training in ecologically valid settings for longer time scales than are currently typically studied and reported.

In a third study addressing oculomotor control, entitled “*Neuromonitoring Correlates of Expertise Level in Surgical Performers: A Systematic Review*” Hannah et al. review the question of how to differentiate novice from experienced surgeons. The authors note that multiple models have been proposed but they all require the subjective analysis of skill and are indirect proxies of expertise. More recently neural imaging-based expertise classification methods have been devised which are inherently objective and outperform current subjective methods of surgical skill evaluation. The authors present a systematic review of the literature in this area identifying both the limitations as well as the benefits of this approach while suggesting future study guidelines to help reliably grow this field, currently in its infancy.

In the article “*Which Comes First in Sports Vision Training: The Software or the Hardware Update? Utility of Electrophysiological Measures in Monitoring Specialized Visual Training in Youth Athletes*” Poltavski et al. compared the order of two types of vision training. Having enrolled 53 youth ice hockey athletes, a cross-over design study was utilized to have half the group perform 5 weeks of Optometric vision therapy, followed by 5 weeks of traditional oculomotor protocols with a second group doing the same tasks in reverse order. An all-in-one, commercial measure of oculomotor performance was used to compare the two groups as well as electrophysiological indexes of EEG and VEP activity. Although both groups improved on all measures of oculomotor performance, there was some evidence noted on electrophysiological testing suggesting that initial vision therapy may be beneficial before traditional oculomotor protocols. Hopefully, the authors in future research will look to move these results from the lab to on-field or on-ice sports performance; to establish an *in-vivo* benefit to the athlete.

The final manuscript entitled “*Egocentric Chunking in the Predictive Brain: A Cognitive Basis of Expert Performance in High-Speed Sports*” by Lappi examines perceptual-cognitive abilities in high-speed sports. Using the motorsport athlete as a paradigm, the authors present a theoretical framework of how perceptual-cognitive abilities may be utilized and evaluated in high-speed sports. The authors review how these abilities are integrated and they propose a “chunking” approach to analyze high speed sports. They note that the chunking approach is applicable to any sport or activity that requires similar perceptual-cognitive demands. The authors suggest that this approach may be useful in translating fundamental research and theory into methods to improve real-world sports performance.

## Author Contributions

DL wrote and edited the Editorial. LA, TH, and DP reviewed and edited the Editorial. All authors contributed to the article and approved the submitted version.

## Funding

LA was supported by United States Army Research Office award W911NF-15-1-0390.

## Conflict of Interest

DL is the owner of ChampionsEdge, LLC. The remaining authors declare that the research was conducted in the absence of any commercial or financial relationships that could be construed as a potential conflict of interest.

## Publisher's Note

All claims expressed in this article are solely those of the authors and do not necessarily represent those of their affiliated organizations, or those of the publisher, the editors and the reviewers. Any product that may be evaluated in this article, or claim that may be made by its manufacturer, is not guaranteed or endorsed by the publisher.

